# Improved fluoroscopy-guided biopsies in the diagnosis of indeterminate biliary strictures: a multi-center retrospective study

**DOI:** 10.1038/s41598-023-39438-2

**Published:** 2023-08-12

**Authors:** Zhe Xiong, Kuangjing Wang, Huahui Zhang, Ying Fang, Fengdong Li, Jin Huang

**Affiliations:** 1 Department of Gastroenterology, Changzhou No.2 People’s Hospital, Changzhou, China; 2 Department of Gastroenterology, The People’s Hospital of Ma Anshan, Ma Anshan, China; 3 Graduate School of Dalian Medical University, Dalian, China; 4 Department of Gastroenterology, The First People’s Hospital of Lianyungang, Lianyungang, China

**Keywords:** Gastroenterology, Medical research

## Abstract

To evaluate the diagnostic accuracy of improved fluoroscopy-guided biopsies for indeterminate biliary strictures (IBDS). A multi-center retrospective study was performed. Patients with IBDS who underwent digital single-operator cholangioscopy (DSOC) and improved fluoroscopy-guided biopsies procedures were included. The individual sensitivity, specificity, and accuracy were analyzed. A total of 67 patients were enrolled in this multi-center retrospective study. The DSOC and improved fluoroscopy-guided biopsies procedures were successfully performed in all cases (100%). The sensitivity, specificity, and accuracy values were 83.3%, 89.5%, and 85.1% for DSOC visual impression; 95.8%, 94.7%, and 95.5% for improved fluoroscopy-guided biopsies procedures, respectively. The sensitivity and accuracy of improved fluoroscopy-guided biopsies were significantly higher compared with DSOC visual impression. Four patients (6.0%, 4/67) occurred adverse events after the procedures. Improved fluoroscopy-guided biopsies had a high diagnostic accuracy of IBDS diagnosis.

## Introduction

Accurate diagnosis of IBDS remains a major challenge. Some benign biliary strictures (BBS) had an overlap in the clinical manifestations with malignant biliary strictures (MBS), such as primary sclerosing cholangitis (PSC) and IgG4-related sclerosing cholangitis. Despite the advanced image technology, it could not accurately distinguish between MBS and BBS^1,2^. Patients with MBS have quite a poor prognosis if it was not diagnosed at the early stage^3^. Based on the data from the previous literature, about 20% of patients with suspect MBS was diagnosed with a benign disease via surgical pathology^1,4–6^. Therefore, making an accurate diagnosis was not only helps to treat patients with MBS in the early stage but also avoids overtreatment in patients with BBS.

Endoscopic retrograde cholangiopancreatography (ERCP) was the most common technology for the diagnose biliary disease. Brushing cytology with ERCP was easy to obtain specimens for the diagnosis, the low sensitivity restricted its promotion^7–9^. ERCP-guided forceps biopsy may have a higher sensitivity compared with brushing cytology because it could provide the deep tissue from the biliary epithelium. However, its sensitivity was still not satisfactory. A meta-analysis had been reported that the two methods combined only had a mild exaltation in the sensitivity^10^.

The peroral cholangioscope (POCS) was used for differentiating between MBS and BBS in recent years. In the early time, mother-baby cholangioscope was used for diagnosing indeterminate biliary strictures (IBDS). Nevertheless, its promotion was limited by difficult operation, expensive, and fragile equipment^11–13^. Digital single-operator cholangioscopy (DSOC) was first reported in 2015. DSOC provided a quality image in the biliary tree and could directly biopsy the site of interest. According to a recent report, DSOC had a low sensitivity for appraisal of IBDS^14^. These results demonstrated that making an accurate diagnosis on IBDS is unreliable only rely on visual impression, forceps biopsies were necessary. However, DSOC-guided biopsies had a low sensitivity for the diagnosis of malignancy^15,16^. The reason may be that the specimens were small and the technology was difficult to operate.

Our previous study demonstrated that the safety and effectiveness of biliary tissue sampling using novel elbow biopsy forceps during ERCP^17^. We had improved the conventional fluoroscopy-guided biopsies. Simply put, improved fluoroscopy-guided biopsies were performed by novel elbow biopsy forceps and with the assistance of DSOC. Our study aimed to assess the diagnostic value of improved fluoroscopy-guided biopsies of IBDS diagnosis.

## Methods

### Patients

Patients with IBDS who underwent the DSOC and improved fluoroscopy-guided biopsies procedures at the Affiliated Changzhou No.2 People’s Hospital of Nanjing Medical University and The People's Hospital of Ma Anshan from August 2018 to March 2021 were enrolled in this retrospective study. The IBDS was defined as: (1) biliary strictures or filling defect of indeterminate nature observed based on the imaging examination such as computed tomography (CT), magnetic resonance imaging (MRI), and endoscopic ultrasonography (EUS); and (2) negative or inconclusive pathological diagnosis after ERCP-guided brushing; but with a suspect MBS. Patients with the follow-up were lost or less than six months after the procedure were excluded.

A total of 67 consecutive patients with IBDS were included in this retrospective study. Clinical data were obtained from the electronic medical record system. All patients had no contradictions to ERCP and DSOC, and all patients submitted their written informed consent before the procedures.

### End points

The primary endpoints were the success rates and the accuracy of diagnosis of the technology program. The successful ERCP and DSOC procedures were defined as the site of the biliary strictures that were observed under the X-ray fluoroscopy and DSOC. The improved fluoroscopy-guided biopsies procedures were considered as successful if the biopsy specimens could be used for histopathological examination. In the case of histopathological diagnosis via biopsy specimens, neoplastic lesions and suspected malignancies were considered malignant, and the rest of histopathological findings were considered benign. The final diagnosis was based on histopathological examination of the surgical specimens or the follow-up no less than six months period by the imaging examination. The second endpoint of the current study was the procedures-related adverse events as recorded in the medical records. The ERCP-related adverse events comprised perforation, bleeding, pancreatitis. DSOC and forceps-related adverse events included cholangitis, biliary bleeding, and biliary perforation.

### Procedures

#### ERCP procedures

All procedures were performed in an endoscopy operating room under anesthetic and sedation patients in the left lateral position. All patients received prophylactic antibiotic treatment. ERCP was performed using a standard therapeutic duodenoscope as usual. In a nutshell, the duodenoscope was delivered the duodenal papilla from the oral cavity. Duodenal papilla cannulation was performed using a CleverCut sphincterotome. The biliary strictures were observed under X-ray fluoroscopy by cholangiography with meglumine diatrizoate. All patients were received endoscopic sphincterotomy and papillary balloon dilation after the successful cholangiography. (If not performed in the past).

#### DSOC procedures

DSOC program was performed using SpyGlass system. The DSOC apparatus was advanced into the bile duct under a wire-guided from the working channel of the therapeutic duodenoscope. The visual impression of biliary strictures obtained by repeated advancement and withdrawal DSOC (Supplementary video 1). Regarding the visual impression, irregular vessels, irregular surfaces, and polypoid tissue were considered malignant. The visual findings of DSOC in each case were evaluated by two experienced endoscopic physicians, one operator and one observer.

#### Improved fluoroscopy-guided biopsies procedures

**Figure 1:**
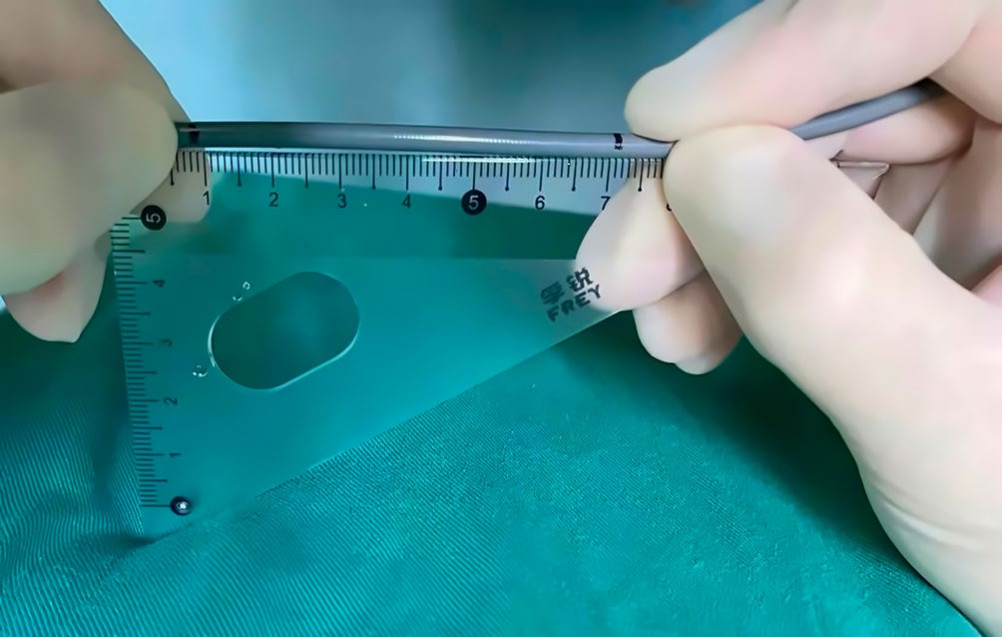
The length of withdrawal DSOC was measured by a ruler at the entrance to the endoscopic channel.

**Figure 2:**
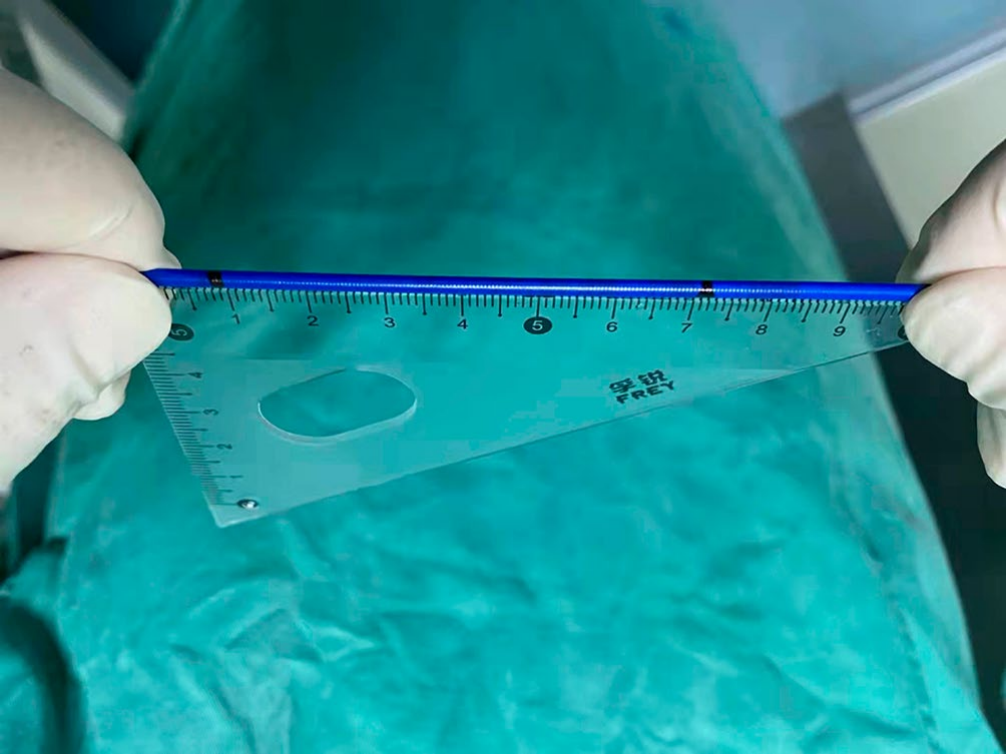
When the tip of the novel elbow biopsy forceps approaches the duodenal papilla, measure the same distance as the length of withdrawal DSOC to make sure that the biopsy procedure was performed at the site of interest.

Improved fluoroscopy-guided biopsies were performed using novel elbow biopsy forceps rather than conventional biopsy forceps. The novel elbow biopsy forceps had described in our previous study^17^. In brief, it had a prebent head of 30 degrees and a wide clamp distance (7.1 mm), which could be easily inserted into the bile duct and obtain adequate tissue sampling. In addition, biopsies at the site of interest were performed with the assistance of DSOC. First, the tip of the DSOC was placed at the site of interest and slowly withdraw the DSOC to the duodenal papilla. Then, the distance from the site of interest to duodenal papilla was confirmed by measuring the length of withdrawal DSOC (Fig. 1). Next, the novel elbow biopsy forceps was advanced to the duodenal papilla, then insert the novel elbow biopsy forceps the same length into the bile duct (Fig. 2). Finally, the site of interest was advanced and biopsied under X-ray fluoroscopy. Biopsies were performed at every stricture no less than 4 times. All procedures were carried out by one of three experienced endoscopic physicians with experience performing > 300/year ERCP.

#### Histopathological examination

All biopsy specimens were fixed in the 10% formalin for pathological evaluation. Histopathological examination was performed by two pathologists with 10 years of experience in bile duct histopathology in two medical centers, respectively. All pathologists know nothing about the patient's clinical data.

#### Statistical analysis

The measurement data are presented as the mean ± standard deviation (SD) or median (range), and the enumeration data are presented as percentages. The accuracy of diagnosis was compared by using the McNemarexact test. *P* < 0.05 was considered significant. Data were analyzed using IBM SPSS.

### Ethical approval

This retrospective study was in accordance with the Declaration of Helsinki and approved by the Ethics Committee of the Affiliated Changzhou No.2 People’s Hospital of Nanjing Medical University. The patients provided their written informed consent to participate in this study.

## Results

### Patients information

From August 2018 to March 2021, 67 consecutive patients were enrolled in this study. The most common clinical manifestations were abdominal pain and jaundice. Among 67 patients, 48 and 19 cases were diagnosed with malignant and benign disease, respectively. 37 patients with MBS were confirmed by surgical specimens, 11 patients with MBS were verified during the follow-up period. The final diagnosis of BBS was confirmed by clinical follow-up in 17 cases, surgical specimens in 2 cases. The median time of the follow-up time was 13 (8–24) months. The location of the stricture, length of the stricture, and the number of biopsies are presented in Table 1.

**Table 1:** Characteristics of the patients.

	N = 67
Age, median (range), years	66 (55–85)
Gender	
Male, n (%)	43 (64.2)
Female, n (%)	24 (35.8)
Clinical presentation, n (%)	
Jaundice	58 (86.6)
Abdominal pain	36 (53.7)
Anorexia	23 (34.3)
Fatigue	18 (26.9)
Weight loss	9 (13.4)
Location of stricture, n (%)	
Hilar	19 (28.4)
Superior	8 (11.9)
Middle	12 (17.9)
Inferior	28 (41.8)
Length of stricture, median (range), mm	16 (8–38)
Number of biopsies, median (range)	5 (4–6)
Final diagnosis, n (%)	
Malignant	48 (71.6)
Benign	19 (28.4)
Biliary stones	8 (42.1)
PSC	6 (31.6)
Chronic pancreatitis	2 (10.5)
Cholecystectomy	3 (15.8)
Following up, median (range), months	13 (8–24)

### Technical success rates and adverse events

All patients have successfully performed ERCP, DSOC, and improved fluoroscopy-guided biopsies procedures. All tissue samples could be used for histological evaluation. In relation to complications, the total incidence rate was 6.0% (4/67). Pancreatitis and cholangitis occurred in two and two cases, respectively. Two cases of mild pancreatitis were significantly improved after somatostatin treatment. Cholangitis occurred in two patients with mild abdominal pain and a maximum temperature of 38.5 °C. The symptoms improved after 5 days of antibiotic treatment. There was no bleeding, perforation, and biliary injury in our study (Table 2).

**Table 2:** Technical success rates and adverse events.

Technical success rates, n/N (%)	
ERCP	67/67 (100)
DSOC	67/67 (100)
Biopsy	67/67 (100)
Adverse events, n (%)	
ERCP-related	
Pancreatitis	2 (3.0)
Others	0 (0)
DSOC and forceps-related	
Cholangitis	2 (3.0)
Biliary bleeding	0 (0)
Biliary perforation	0 (0)

### DSOC visual and improved fluoroscopy-guided biopsies evaluation

**Figure 3:**
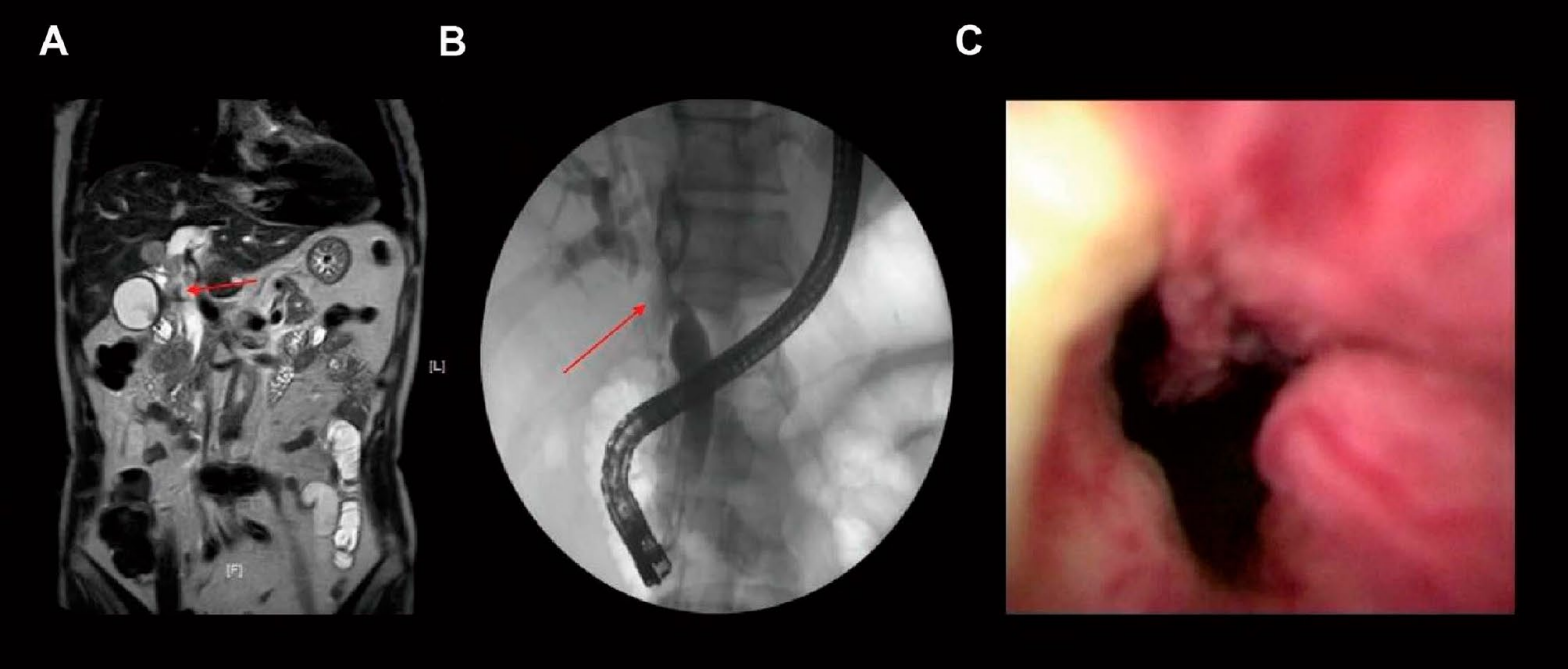
(**A**) MECP image of malignant biliary stricture with an arrow suggesting the site of stricture. (**B**) Cholangiography of malignant biliary stricture with an arrow suggesting the site of stricture. (**C**) DSOC image of malignant biliary stricture.

DSOC procedures were performed in all cases, among them 42 (62.7%) patients were diagnosed as malignant and 25 (37.3%) were diagnosed with benign disease. Figure 3 shows representative images of MRCP, cholangiography and DSOC for malignant biliary strictures. The sensitivity, specificity, and accuracy for DSOC visual assessment were 83.3%, 89.5%, and 85.1%. Based on biopsies histopathology, 47 (70.1%) cases were diagnosed as malignant and the remaining patients were diagnosed with benign disease. The improved fluoroscopy-guided biopsies procedures had a 95.8% sensitivity, a 94.7% specificity, and a 95.5% accuracy, respectively. Improved fluoroscopy-guided biopsies procedures had a higher sensitivity (95.8% vs. 83.3, *P* = 0.031) and overall accuracy (95.5% vs. 85.1%, *P* = 0.039) for diagnosing IBDS compared with DSOC visual impression (Table 3).

**Table 3:** Performance characteristics of two methods in patients with IBDS (n = 67).

(%)	Improved fluoroscopy -guided biopsies (%)	DSOC visual impression (%)	*P* value
Sensitivity	95.8	83.3	0.031
Specificity	94.7	89.5	NS
Accuracy	95.5	85.1	0.039

NS: No significance.

## Discussion

In this study, all patients have successfully performed ERCP, DSOC, and improved fluoroscopy-guided biopsies procedures. The use of DSOC visual impression for distinguishing between MBS and BBS had a high sensitivity (83.3%) and accuracy (85.1%) of IBDS diagnosis. Improved fluoroscopy-guided biopsies procedures were performed under ERCP and with the assistance of DSOC, unlike as usual. Improved fluoroscopy-guided biopsies procedures had a higher sensitivity and accuracy for diagnosing IBDS compared with the previous study^10^. In addition to this, our study demonstrated that the sensitivity (95.8%) and accuracy (95.5%) of the improved fluoroscopy-guided biopsies procedures achieved a significant exaltation compared with DSOC visual impressions.

Despite the development of non-invasive imaging examination, accurate identification of the characterization of IBDS was still difficult. POCS had significant advantages for the visualization of the biliary tree compared with ERCP. In the early, dual-operator mother-baby cholangioscopy was used in clinical practice. However, it had some disadvantages, such as, operate difficulty, low-quality image, and a small working channel^18,19^. The DSOC had overcome these shortcomings, it only required an experienced endoscopist to operate and could provide a high-quality image in the biliary tree^7,20^. Based on the data from recent literature, the sensitivity and specificity of DSOC visual impression for diagnosing the IBDS were 83–97% and 67–96%, respectively^7,21–23^. The sensitivity and specificity of DSOC visual impression were 83.3% and 89.5% in the current study, which is similar to these results. However, recent research reported the DSOC visual impression had a low sensitivity for IBDS. A retrospective single-center study reported that the sensitivity for DSOC visual impression was 64%^15^. The low sensitivity maybe it was because many patients (40%) had PSC, which was quite difficult to differentiate between MBS and BBS^15,24,25^. Neovascularization was considered as a maker of MBS in POCS visual impression. However, their results show that neovascularization could be observed in many patients with biliary stents in situ^15^. Similarly, another report shows a low sensitivity for DSOC visual impressions of IBDS and had a considerable interobserver variation, in which, about 27% of patients with PSC, but the diagnostic accuracy had no significant difference even if excluded the patients with PSC^14^. Therefore, accurate diagnosis of IBDS can not only rely on DSOC visual impression, and biopsies are necessary.

DSOC-guided biopsies were widely used for obtaining biliary tissue samples. A recent Meta-Analysis study, which involved 356 patients showed that the pooled sensitivity for MBS was 74%^4^. However, DSOC-guided biopsies had some shortcomings. First, the tissue samples were small because the working channel of DSOC is small (1.2 mm). And biopsies at the site of interest sometimes were quite difficult to perform even for experienced endoscopists. Biopsy under direct POCS may obtain more adequate samples than DSOC-guided biopsies because direct POCS had a larger accessory channel^26^. However, direct POCS procedures were performed more difficult than ERCP and DSOC procedures. A previous study showed that biopsies under ERCP and direct POC had no difference in making a distinction between MBS and BBS^2^. The development of an effective bile duct biopsy program was extremely important for clinical practice. Thus, we designed improved fluoroscopy-guided biopsies procedures. Unlike conventional fluoroscopy-guided biopsies, it was performed using novel elbow biopsy forceps. Conventional biopsy forceps was difficult to approach the bile duct and obtain adequate biliary tissue because the head of conventional biopsy forceps ran parallel to the bile duct. Novel elbow biopsy forceps could overcome these shortcomings benefit from its prebent head and wide distance of clamp (7.1 mm). Furthermore, target biopsies at the site of interest sometimes were quite difficult to perform under ERCP. To overcome this disadvantage, we use the DSOC to accurately locate the site of interest. This not only reduced radiation exposure to both the operator and patient^27^, but also the borders of the lesion were more accurately observed than cholangiography. The distance from the duodenal papilla to the site of interest was measured with the assistance of DSOC. The novel elbow biopsy forceps was advanced and approached the site of interest refer to this distance. This method makes sure that accurate biopsies at the site of interest and tissue adequacy under X-ray fluoroscopy. In fact, the diagnostic yield of improved fluoroscopy-guided biopsies was higher than conventional fluoroscopy-guided biopsies^10^. Intraductal ultrasonography (IDUS)-guided biopsies could biopsy the strictures in real-time. A recent prospective study reported that IDUS-guided biopsies had a higher diagnostic accuracy compared with conventional fluoroscopy-guided biopsies (90.8% vs 76.9%), which was similar to our result^28^. IDUS-guided biopsies were performed by IDUS probe and biopsy forceps together in the working channel of the duodenoscope, which undoubtedly increased the difficulty in tissues sampling, and IDUS probe may lead to biliary trauma. In the current study, we further found that improved fluoroscopy-guided biopsies had a higher sensitivity and accuracy compared with DOSC visual impression. It points out that when it was hard to discriminate between MBS and BBS using DSOC, improved fluoroscopy-guided biopsies may be applied for it.

Pancreatitis was the most common adverse event after ERCP, and the incidence was range 3.9% to 10.1% based on recent reports^29,30^, which was similar to our study (pancreatitis occurred in two cases). In our study cholangitis appeared in two cases (2/67, 3.0%). According to the recent report that the incidence of cholangitis after the DSOC procedure was range from 3.1% to 7.5%^31,32^, which was consistent with our findings. Antibiotic prophylaxis may reduce the incidence of cholangitis. All patients received a treatment of antibiotics before procedures in our study. There was no evidence of biliary bleeding or perforation after improved fluoroscopy-guided biopsies procedures in the current study.

There were some limitations in this study. This is a small sample and retrospective study even if data is obtained from two medical centers. Another limitation of this study was that endoscopists were not blinded to the clinical information of the patients, which may have influenced their diagnosis. In the future, multicenter, large sample, prospective studies need to validate the result of our study.

## Conclusion

Taken together, improved fluoroscopy-guided biopsies procedures were a safe and effective method for IBDS. In IBDS, improved fluoroscopy-guided biopsies procedures had a high diagnostic yield and should be considered for IBDS.

### Supplementary Information

Supplementary Information 1.

Supplementary Video 1.

## Data Availability

The datasets used or analyzed during the current study are available from the corresponding author on reasonable request.
