# Supplementary material for: Improved fluoroscopy-guided biopsies in the diagnosis of indeterminate biliary strictures: a multi-center retrospective study
Source: Sci Rep. 2023 Aug 12;13:13152. doi: 10.1038/s41598-023-39438-2 (PMC10423265; doi:10.1038/s41598-023-39438-2)
Supplement: Supplementary file 1 — Supplementary Information 1. [file 41598_2023_39438_MOESM1_ESM.docx]

Supplementary video 1. Adenomatous hyperplasia in the bile duct were observed by DSOC.
